# Mitigation of Malaria in Sub-Saharan Africa through Vaccination: A Budding Road Map for Global Malaria Eradication

**DOI:** 10.4314/ejhs.v35i3.9

**Published:** 2025-05

**Authors:** Esther Ugo Alum, Christine Ainebyoona, Chinedu Ogbonnia Egwu, Hope Onohuean, Okechukwu Paul-Chima Ugwu, Daniel Ejim Uti, Benedict Nnachi Alum, Darlington Arinze Echegu

**Affiliations:** 1 Department of Research and Publications, Kampala International University, Main Campus, P. O. Box 20000, Kampala, Uganda; 2 Faculty of Business and Management Sciences, Kampala International University, Kampala Uganda; 3 Department of Medical Biochemistry, Faculty of Basic Medical Sciences, College of Medical Sciences, Alex Ekwueme Federal University, Ndufu-Alike, Ikwo, Ebonyi State, Nigeria; 4 Department of Pharmacology and Toxicology, School of Pharmacy Kampala International University Western Campus Ishaka, Uganda

**Keywords:** Malaria, Sub-Saharan Africa, Malaria vaccine, RTS,S/AS01, Plasmodium falciparum, SDG 3

## Abstract

**Background:**

Malaria remains a major public health and economic burden globally, with sub-Saharan Africa (SSA) accounting for over 90% of cases and deaths. Children under five and pregnant women are the most vulnerable. Resistance to artemisinin-based therapies and insecticides has compounded the challenge. This review evaluates malaria mitigation strategies, emphasizing the potential of vaccination, particularly RTS,S/AS01, as a cornerstone for malaria eradication and achievement of Sustainable Development Goal 3 (SDG 3).

**Methods:**

A narrative review approach was adopted. Peer-reviewed literature from 2014 to 2024 was sourced from credible scientific databases, including PubMed, Google Scholar, and ScienceDirect. Selection prioritized studies on malaria control strategies, vaccine trials, efficacy data, implementation frameworks, and health system challenges specific to SSA. The review critically analyzed vaccine trials, community perceptions, and logistical concerns while incorporating the authors' expert perspective.

**Results:**

The RTS,S/AS01 vaccine demonstrated moderate efficacy (30–55%) in preventing malaria in children under five in Ghana, Kenya, and Malawi. Other candidates such as R21/Matrix-M showed up to 77% efficacy in trials. Implementation challenges include limited funding, vaccine hesitancy, poor healthcare infrastructure, and community misconceptions. However, integration with routine immunization, enhanced community engagement, and increased investment in health systems significantly improve vaccine acceptance and impact.

**Conclusion:**

Vaccination represents a promising, cost-effective, and scalable strategy for malaria eradication in SSA. Strengthening healthcare infrastructure, amplifying vaccine acceptance campaigns, and increasing funding for research and vaccine production are pivotal. Malaria vaccination offers a practical and sustainable pathway toward global malaria elimination and the realization of SDG 3 by 2030.

## Introduction

Malaria remains one of the top ten causes of premature death and one of the most life-threatening communicable diseases globally (1) It is caused by protozoan parasites, commonly known as *Plasmodium*. While there are rare instances of malaria transmission through blood transfusion, mother-to-fetus transmission, and organ transplants, the primary mode of transmission is through the bite of infected female Anopheles mosquitoes, which release the parasite into the bloodstream. A particular strain of *Plasmodium, P. knowlesi*, can also be transmitted zoonotically from monkeys to humans via mosquitoes (2, 3). *Plasmodium* is classified into various species, including *P. falciparum, P. malariae, P. vivax, P. ovale*, and *P. knowlesi* (2, 3). Among these, *P. falciparum* and *P. vivax* are the most dangerous, with *P. falciparum* responsible for more than 90% of global malaria cases (2). The incubation period for malaria varies depending on the parasite species; for example, *P. falciparum* has an incubation period of 9-14 days, while *P. vivax* and *P. ovale* last for 12-18 days, and *P. malariae* can last 18-40 days before symptoms appear (4).

Malaria symptoms include chills, fatigue, headache, fever, nausea, and vomiting. Severe malaria can lead to organ damage (5), and anemia is especially pronounced during malaria infection, making children and pregnant women particularly vulnerable (6). Diagnosis is typically done through microscopic blood examination or other rapid diagnostic tests, such as polymerase chain reaction (PCR) (1).

Malaria is a significant global health challenge, particularly in the Eastern Mediterranean, Western Pacific, Southeast Asia, and Sub-Saharan Africa (SSA). However, SSA accounts for over 95% of the global malaria burden. According to the WHO Malaria Report, in 2021, 247 million people were infected with malaria, and around 619,000 deaths occurred (1). In SSA, countries such as Nigeria, the Democratic Republic of Congo, Uganda, and Mozambique accounted for nearly 50% of the global malaria cases in 2021 (7). In 2019, countries like Nigeria, Burkina Faso, Liberia, Niger, Benin, Togo, Mozambique, Ivory Coast, and Sierra Leone had the highest number of malaria-related deaths (8), reinforcing the fact that SSA is the epicenter of the disease. Notably, SSA contributes to about 96% of all malaria-related deaths globally, with children under five being the most affected. In SSA, a child under five dies from malaria every two minutes (9). In 2019, over 12 million pregnant women in SSA contracted malaria, leading to complications such as miscarriages, low birth weight, and severe anemia in newborns (7).

Malaria not only impedes economic growth but also places a considerable strain on healthcare systems and worsens the impact of non-communicable diseases such as gestational hypertension and heart failure. Malaria is also a significant barrier to achieving the United Nations' Sustainable Development Goal 3 (SDG3), which aims to reduce malaria incidence and deaths by 90% by 2030. Vaccination has emerged as a promising strategy, especially since it is cost-effective and targets the most vulnerable population—children under five. To compile effective malaria control measures for this review, we explored several credible databases for articles published between 2014 and 2024. It is noteworthy that vaccinating children under five years old has been identified as one of the most effective methods for eradicating malaria. Therefore, the malaria vaccine not only supports the realization of SDG3—ensuring healthy lives and well-being for all—but also serves as a key tool in the global effort to eliminate malaria.

## Methodology

This narrative review was conducted by combining relevant published literature from 2014 to 2024. A comprehensive search was performed across multiple reputable databases including PubMed, Google Scholar, ScienceDirect, and WHO publications. Keywords such as “malaria vaccine,” “sub-Saharan Africa,” “Plasmodium falciparum,” “RTS,S/AS01,” “malaria mitigation,” and “Sustainable Development Goal 3” were used to retrieve pertinent articles. The selection criteria emphasized peer-reviewed original research, systematic reviews, clinical trial data, and global and regional health reports focusing on malaria vaccination strategies, efficacy, and implementation challenges in sub-Saharan Africa. All retrieved sources were critically assessed for relevance and quality. The evidence gathered was collaboratively analyzed by the authors to highlight the role of vaccination as a promising strategy for malaria mitigation, especially in children under five years of age-the most affected demographic.

**Factors militating against malaria mitigation in SSA**: Despite global attention and funding dedicated to malaria elimination in SSA, the disease continues to persist and has even become more severe in recent years. Several factors contribute to the ongoing challenge. First, the climate in SSA is highly favorable for mosquito breeding, as mosquitoes require stagnant water, which is abundant due to regular rainfall. The region's warm temperatures further support mosquito reproduction (10).

Second, inadequate healthcare resources, including a shortage of healthcare workers and underfunded healthcare systems, contribute to the high malaria prevalence in the region. As a result, many residents turn to traditional medicine, which often relies on unrefined plant-based materials. While plants have therapeutic potential, such as *Sphenocentrum jollyanum* (4) and *Buchholzia coriacea* (11), these remedies are not as effective as conventional antimalarial drugs and may cause harmful side effects if improperly dosed. The growing resistance of malaria parasites to artemisinin-based combination therapy (ACT) and the increasing resistance of mosquitoes to insecticides have further complicated malaria control efforts.

Additionally, malaria is often associated with poverty, which in turn leads to poor housing conditions, malnutrition, and weakened immunity. Many individuals in SSA cannot afford antimalarial medications or preventive measures such as insecticide-treated bed nets and indoor insecticides (12).

**Malaria mitigation in SSA and achievement of sustainable development Goal (SDG 3)**: The United Nations has incorporated the fight against malaria into its core agenda through SDG 3, which aims to reduce malaria incidence and deaths by at least 90% and eliminate the disease in at least 35 of the most affected countries by 2030. To achieve this, several malaria control mechanisms have been implemented, including vector control strategies such as the use of insecticide-treated bed nets, indoor insecticide spraying, chemoprevention with antimalarial drugs, and the use of artemisinin-based combination therapies (ACT) to treat infected individuals (13).

Malaria continues to pose a significant health burden in SSA, straining already fragile healthcare systems and impeding economic growth, thus hindering the achievement of SDG 3. The disease also worsens the severity of other conditions, particularly non-communicable diseases. For instance, studies have shown a correlation between malaria and heart failure (14), and malaria during pregnancy has been linked to gestational hypertension (15). Therefore, eliminating malaria in SSA is critical to achieving the goal of ensuring healthy lives and well-being for all by 2030.

The recent development of resistance to ACT and insecticides used for mosquito control has raised concerns in the battle against malaria. Strengthening malaria prevention measures and investing in research to develop more effective antimalarial drugs and vaccines are essential for achieving SDG 3.

**Malaria vaccines, reported trials, and level of success in sub-Saharan Africa**: Recent research highlights the challenges and progress in developing a malaria vaccine specifically for the sub-Saharan Africa region. The RTS,S/AS01 vaccine has shown promise but faces significant obstacles in its deployment, such as limited community involvement and concerns about potential adverse effects (31). A scoping assessment revealed numerous potential malaria vaccines, with R21 exhibiting the highest effectiveness at 77% over a 12-month period, compared to RTS,S, which has an efficacy of 55% (32). However, the decreasing effectiveness of RTS,S, which drops to 60-70% after 18 months, remains a concern (33).

Malaria control also faces other significant challenges, including the growing resistance of *Plasmodium* parasites to artemisinin-based treatments and the increasing resistance of mosquito vectors to pesticides (33). Despite these challenges, there is optimism surrounding the development of new vaccines, as several promising candidates are currently under investigation (34). To ensure successful vaccine adoption, it is crucial to address community perceptions, improve vaccine effectiveness, and ensure broad availability across African communities (32).

**Strengthening research as a tool for malaria mitigation goals**: Extensive research and innovation are required to achieve a 90% reduction in malaria by 2030, in line with Sustainable Development Goal 3 (SDG 3). Unfortunately, malaria research in Sub-Saharan Africa (SSA), the most affected region, does not match the disease's pervasiveness in this area (43). Therefore, it is essential to amplify malaria research efforts by increasing funding from both the government and private sectors. Regrettably, some SSA countries receive very limited malaria funding, which hampers the progress of malaria elimination (44).

Malaria research should prioritize the development of new antimalarial drugs and insecticides, as the *Plasmodium* parasites have developed resistance to artemisinin and pyrethroids. In addition, research should focus on strengthening vector control measures, vaccine development, and enhancing malaria diagnostic tools (44). A robust research base is critical to achieving the targeted malaria eradication by 2030.

**Malaria pervasiveness in children and malaria vaccines**: Malaria is a lethal disease that is treatable and, most importantly, preventable. Children, especially those under five, are the most vulnerable. In SSA, a child under five dies every two minutes from malaria (11). Of the 409,000 malaria deaths in 2019, 274,000 were among children under five (45). In 2021, 80% of malaria-related deaths in SSA occurred in children under five years old (33). Therefore, strengthening malaria elimination strategies targeted at children is of paramount importance.

After three decades of extensive collaboration between GlaxoSmithKline (GSK), the Program for Appropriate Technology in Health (PATH), and several African research centers, a malaria vaccine has been approved by the World Health Organization (WHO) for children in most malaria-endemic regions (46). The RTS,S/AS01 vaccine (also called Mosquirix) targets *Plasmodium falciparum*, the deadliest and most prevalent strain in SSA. Pilot studies were conducted in Ghana, Kenya, and Malawi, where about 2.3 million children have been vaccinated. The vaccine has led to a significant reduction in malaria cases in these countries, and it has been shown to be safe (47).

The malaria vaccine schedule involves four doses, starting at five months of age, through routine immunization. The first three doses are given at four-week intervals, completed by nine months of age, and the last dose is administered at 15–18 months. Data from the pilot countries show that more than two-thirds of vaccinated children experienced a significant reduction in malaria incidence. Importantly, the malaria vaccine is cost-effective compared to other antimalarial measures (47). Thus, the feasibility, availability, and acceptability of the malaria vaccine must be strengthened, as it has proven to be effective in malaria control.

**Embracing the malaria vaccine- The roadmap to global malaria eradication and achievement of SDG 3**: Following the introduction of RTS,S in 2021 in three malaria-endemic regions of SSA, reports of significant improvements in malaria control have led to a higher demand for the vaccine than its current supply can meet (48). Lack of funding has impeded vaccine production to meet this demand, making it crucial to boost funding for vaccine production and research into other malaria vaccines.

Interestingly, research is ongoing to develop two additional malaria vaccines targeting the sexual stage of the malaria parasite and its messenger ribonucleic acid (mRNA), both aimed at blocking parasite transmission. Strengthening the funding for these vaccine developments will play a crucial role in achieving malaria eradication by 2030.

Despite the improvement in malaria control with the use of RTS,S, it still faces partial acceptance in SSA. Therefore, amplifying campaigns to improve vaccine acceptance is essential. Vaccine reluctance, common in low- and middle-income countries, has hindered the elimination of diseases like polio in regions such as Pakistan and Nigeria (49-51). However, the reversal of polio outbreaks through improved vaccine campaigns demonstrates that similar efforts could help improve malaria vaccine acceptance in SSA. Notably, malaria vaccine acceptance rates have been high in some SSA countries: 84.2% in Tanzania (52), 91.6% in Nigeria (53), 88% in Kenya (49), and 97% in Sierra Leone (54).

Nevertheless, challenges remain. In Ethiopia, for instance, low vaccine acceptance (32.3%) was attributed to fears that the vaccine could paralyze children (50). In Ghana, misinformation and misconceptions about the vaccine contributed to low acceptance (55). Cultural and religious practices in SSA also sometimes perceive vaccination as harmful, further complicating vaccine uptake. Involving community and religious leaders in malaria vaccine campaigns can help dispel these misconceptions.

Additionally, grassroots education about malaria as a threat is crucial for improving vaccine acceptance. Community-based campaigns, like those used in HIV/AIDS prevention, can play a significant role in enhancing vaccine uptake (56). Moreover, improving healthcare facilities and increasing the number of health workers is essential for supporting vaccine delivery. Since malaria is intertwined with poverty, individuals in lower-income areas may face difficulties accessing vaccines, even if they are free. Thus, increasing the number of healthcare centers, particularly in rural areas, is vital. Governments and other supporting bodies should fund vaccine production and ensure its free distribution.

Co-administration of the malaria vaccine with other routine vaccines, such as Vitamin A, yellow fever, measles, rubella, and scabies, has not resulted in adverse health outcomes (57). Although the vaccine's efficacy is not yet optimal, its clinical prospects remain promising. According to phase 3 results, RTS,S provides 1,744 malaria prevention cases for every 1,000 infants vaccinated. The vaccine also prevents 116,480 malaria cases and 484 deaths per 100,000 vaccinated infants. Importantly, RTS,S is far cheaper than other malaria prevention measures (58, 59).

**Implementation of the malaria vaccine in Sub-Saharan Africa**: Malaria remains a significant public health issue in Sub-Saharan Africa, causing hundreds of thousands of deaths annually, primarily among children under five. The introduction of the malaria vaccine represents a major step forward in the global fight against the disease (35). However, implementing the vaccine in resource-limited settings requires careful planning and coordination.

**Vaccine development and efficacy**: RTS,S/AS01, also known as Mosquirix, developed by GlaxoSmithKline (GSK) in collaboration with the PATH Malaria Vaccine Initiative and funded by the Bill & Melinda Gates Foundation, has undergone extensive clinical testing in various African countries (60). While the vaccine offers partial protection against malaria in young children, with effectiveness rates around 30-40%, it still represents a significant reduction in malaria's impact, particularly when combined with other prevention strategies like insecticide-treated bed nets and antimalarial medications.

**Pre-implementation Planning-Strengthening health systems**: Successful vaccine delivery requires enhancing Sub-Saharan Africa's healthcare infrastructure. This includes building facilities, training healthcare personnel, and improving the efficiency of the healthcare system (31). Adequate infrastructure is necessary to store and transport vaccines, especially in rural areas (61, 62). Capacity building is essential to train healthcare workers in vaccine administration, recognizing adverse reactions, and educating communities about the vaccine's benefits (63).

**Community engagement and demand creation**: The success of the malaria vaccine implementation relies on local community acceptance. Vaccine hesitancy, driven by misinformation, cultural beliefs, and distrust in healthcare systems, must be addressed through public health campaigns. These efforts should use media channels such as radio, television, and social media, as well as community outreach activities, to educate the public about the vaccine's advantages (31, 64). Engaging communities in decision-making can foster trust and ensure the vaccine meets local needs.

**Pilot implementation and monitoring**: Pilot programs in specific regions should be used to identify potential challenges and areas for improvement. These pilots will help refine strategies for broader implementation, with monitoring and evaluation systems to assess vaccine effectiveness, coverage, and any adverse effects (65, 66).

**Scaling up- National and regional rollout**: Once pilot projects have been completed, scaling up the vaccine rollout will require cooperation among national governments, international organizations, and donors (67, 68). Incorporating the malaria vaccine into national vaccination programs can optimize distribution and reduce costs. Long-term financial support is essential, and engaging in regional cooperation will help enhance efforts to combat malaria across SSA.

**Addressing challenges and ensuring sustainability**: Challenges to vaccine implementation include reluctance to vaccinate, logistical hurdles, and the emergence of vaccine-resistant malaria strains. Continued community engagement, robust surveillance systems, and equitable vaccine distribution are crucial for overcoming these obstacles (31). Education campaigns and efficient distribution networks, particularly in marginalized areas, are essential for ensuring broad vaccine uptake (69).

**Long-term impact and future directions**: The malaria vaccine has the potential to significantly reduce malaria's impact in Sub-Saharan Africa, saving countless lives. However, ongoing research is necessary to develop more effective vaccines, integrate new vaccine technologies like mRNA platforms, and improve malaria control strategies. The successful implementation of the malaria vaccine in SSA represents a major step toward global malaria eradication, contributing significantly to the achievement of SDG 3.

## Conclusion

Malaria remains a global health and economic burden, with Sub-Saharan Africa being the epicenter of the disease. Children under five are particularly vulnerable. Malaria exacerbates the severity of other diseases, making its elimination crucial for overall health improvement. The malaria vaccine, particularly for children under five, offers a cost-effective strategy compared to other interventions and has become a promising tool for malaria eradication. To achieve malaria elimination by 2030, it is essential to increase funding for vaccine production, improve vaccine acceptance, strengthen healthcare systems, and invest in malaria research in Sub-Saharan Africa.

## Figures and Tables

**Table 1:** Current Malaria Control Programs, their Shortcomings, and How the Malaria Vaccine Fits into Malaria Mitigation

S/NO	Malaria control program	Short coming	Role of malaria vaccine in mitigation	References
**1**	Insecticide-Treated Nets (ITNs)	The emergence of pesticide resistance in mosquito populations, coupled with inadequate coverage in rural regions and the inappropriate usage and maintenance of nets.	The malaria vaccination can offer supplementary safeguarding, particularly in areas with significant insecticide-treated net (ITN) resistance, hence diminishing total transmission.	(16)
**2**	Indoor Residual Spraying (IRS)	Challenges include resistance to insecticides, expensive operational expenses, and environmental problems related to the use of chemicals.	The vaccination provides a supplementary strategy, diminishing dependence on pesticides and conferring protection to humans even in regions with resistant insects.	(17, 18)
**3**	Antimalarial Drugs (Prophylaxis and Treatment)	Challenges in the field of drug resistance, such as resistance to chloroquine and artemisinin, limited availability in distant regions, and problems related to side effects and patient adherence.	The vaccine has the potential to mitigate the occurrence of malaria, hence diminishing the necessity for treatment and alleviating the burden on the emergence of drug resistance.	(19)
**4**	Larval Source Management (LSM)	Demands continuous involvement from the community; efficacy is contingent upon specific local circumstances; poses difficulties in regions with a high number of breeding grounds.	Although LSM can decrease mosquito populations, the vaccination can provide protection to persons against any remaining transmission, particularly in regions where LSM implementation is challenging.	(20)
**5**	Health Education and Behavioral Change Programs	The efficacy of the variable is influenced by cultural barriers and disinformation, necessitating ongoing dedication of time and money.	The vaccination can enhance the effectiveness of teaching initiatives by offering concrete protection and promoting the adoption of other preventative measures.	(21)
**6**	Rapid Diagnostic Tests (RDTs) and Surveillance	Uneven availability of reliable testing; possibility of incorrect negative results; delays in starting therapy in areas with inadequate resources.	The vaccine has the potential to decrease the occurrence of severe cases, so alleviating the strain on diagnostic and treatment systems, and enabling more efficient surveillance.	(22)
**7**	Mass Drug Administration (MDA)	The risk of resistance is high and addressing it needs a significant amount of infrastructure and resources. However, this approach is not viable in the long run.	The vaccine has the potential to decrease the frequency of Mass Drug Administration (MDA) campaigns required, hence promoting sustainable long-term control by lowering transmission rates.	(23)

**Table 2:** Key statistical evidence from clinical trials of malaria vaccines, challenges encountered, and current strategies to improve vaccine efficacy

S/NO	Vaccine	Phase	Efficacy	Sample size	Challenges	Current strategies	References
**1**	RTS,S/AS01	Phase III	~30-50% efficacy in children under 5 years	15,460 children	The protection provided has a limited time and the effectiveness might vary.	Administration of additional doses to enhance the effectiveness of a vaccine, in conjunction with other methods of preventing and managing malaria.	(23)
**2**	PfSPZ Vaccine	Phase I/II	~80-100% efficacy in controlled human malaria infection (CHMI)	40 adults	Expensive production expenses and difficulties in managing logistics	Utilizing genetic alteration to enhance effectiveness and refining ways of administration.	(24)
**3**	ChAd63/MVA ME-TRAP	Phase IIb	67.7% efficacy in 6-10 weeks	411 children	Limited efficacy in field conditions, durability	Investigating prime-boost strategies,	(25)
**4**	R21/Matrix-M	Phase IIb	77% efficacy in 1-year follow-up	450 children	Possible decline of immunity over a period of time	Additional doses to enhance immunity, highly developed substances that enhance the effectiveness of vaccines	(26)
**5**	SPf66	Phase III	31% efficacy in children	20,000 people	Ineffectiveness observed across different age cohorts	Direct attention towards the development of multistage vaccinations and the improvement of adjuvants.	(27)
**6**	GMZ2	Phase IIb	14% efficacy in 1-year follow-up	1,019 children	Low immunogenicity	Enhancing the antigenic composition and integrating with other vaccinations	(28)
**7**	CS/NANP-Repeat Antigen	Phase II	50-60% efficacy	50 adults	Transient immunity, diverse reactions	Utilizing nanoparticles for delivery and optimizing adjuvants.	(29)
**8**	PfRH5-based vaccines	Phase I/IIa	~33-50% efficacy in CHMI	24 adults	Requirement for increased immunogenicity	Researching fusion proteins and improving techniques for delivering vaccines.	(30)

**Table 3:** Malaria Vaccines Efficacy in Sub-Saharan Africa

S/NO	Vaccine	Country tested in sub-Saharan Africa	Level of success	Reference
**1**	RTS,S/AS01	Ghana, Kenya, Malawi	The initial authorized malaria vaccine. The effectiveness of the vaccine is around 39% after four doses, and it can reach up to 55% in regions where malaria is seasonal. Decreases the number of severe malaria cases by approximately 30% in young children.	(35)
**2**	PfSPZ Vaccine	Mali, Tanzania	Demonstrated a 29% level of protection against malaria in Mali and a 52% effectiveness in lowering the occurrence of malaria in adults in Tanzania.	(36)
**3**	ChAd63/MVA ME-TRAP	Burkina Faso, The Gambia	Exhibited a 67% effectiveness in Controlled Human Malaria Infection (CHMI) experiments, but has not demonstrated significant effectiveness in real-world field research.	(36, 37)
**4**	R21/Matrix-M	Burkina Faso	The reported efficacy over a period of 12 months was 77%, which exceeded the World Health Organization's target of 75% efficacy.	(38)
**5**	SPf66	Tanzania, The Gambia	Originally, the vaccine demonstrated an efficiency of around 31%. However, further extensive studies conducted in sub-Saharan Africa revealed limited to no effectiveness, which ultimately resulted in its discontinuation.	(39)
**6**	GMZ2	Burkina Faso, Gabon	Demonstrated limited effectiveness, with a slight decrease of around 14% of clinical malaria episodes, during Phase II studies. This level of efficacy was considered inadequate to justify continued research.	(40)
**7**	CS/NANP-Repeat Antigen	No large-scale trials reported	Initial phase of development. Demonstrates promise in stimulating immunological responses, but, there is still a lack of thorough effectiveness evidence specifically from sub-Saharan Africa.	(41)
**8**	PfRH5-based vaccines	Not yet extensively tested in sub-Saharan Africa	Specifically engineered to selectively bind to the blood-stage antigen PfRH5. Preliminary trials indicate encouraging levels of immunogenicity, but, comprehensive effectiveness trials on a wide scale in sub-Saharan Africa are still awaiting completion.	(42)

## References

[1] The Lancet Microbe (2022). Malaria progress stumbles, but new tools offer hope. The Lancet Microbe.

[2] Egwu CO, Aloke C, Chukwu J, Agwu A, Alum E, Tsamesidis I (2022). A world free of malaria: It is time for Africa to actively champion and take leadership of elimination and eradication strategies. Afr Health Sci.

[3] Alum EU, Tufail T, Agu PC, Akinloye DI, Obaroh IO (2024). Malaria pervasiveness in Sub-Saharan Africa: Overcoming the scuffle. Medicine.

[4] Ekpono EU, Aja PM, Ibiam UA, Alum EU, Ekpono UE (2019). Ethanol Root-extract of Sphenocentrum jollyanum Restored Altered Haematological Markers in Plasmodium berghei-infected Mice. Earthline Journal of Chemical Sciences.

[5] Egwu CO, Aloke C, Chukwu J, Nwankwo JC, Irem C, Nwagu KE (2023). Assessment of the Antimalarial Treatment Failure in Ebonyi State, Southeast Nigeria. Journal of Xenobiotics.

[6] Obeagu EI, Nimo OM, Bunu UM, Ugwu OPC, Alum EU (2023). Anaemia in children under five years: African perspectives. International Journal of Current Research in Biology and Medicine.

[7] Fact sheet about malaria.

[8] Li Z, Shi J, Li N, Wang M, Jin Y, Zheng Z (2022). Temporal trends in the burden of non-communicable diseases in countries with the highest malaria burden, 1990–2019: Evaluating the double burden of non-communicable and communicable diseases in epidemiological transition. Globalization and Health.

[9] Childhood diseases | UNICEF https://www.unicef.org/health/childhood-diseases.

[10] Alum EU, Ugwu OPC, Egba SI, Uti DE, Alum BN (2024). Climate Variability and Malaria Transmission: Unraveling the Complex Relationship. INOSR Scientific Research.

[11] Enechi OC, Okagu IU, Amah CC, Ononiwu PC, Igwe JF, Onyekaozulu CR (2021). Flavonoid-rich extract of *Buchholzia coriacea* Engl. seeds reverses *Plasmodium berghei*-modified haematological and biochemical status in mice. Scientific African.

[12] Sarfo JO, Amoadu M, Kordorwu PY, Adams AK, Gyan TB, Osman AG (2023). Malaria amongst children under five in sub-Saharan Africa: a scoping review of prevalence, risk factors and preventive interventions. Eur J Med Res.

[13] World Health Organization (2018). Towards a global action plan for healthy lives and well-being for all: uniting to accelerate progress towards the health-related SDGs.

[14] Brainin P, Mohr GH, Modin D, Claggett B, Silvestre OM, Shah A (2021). Heart failure associated with imported malaria: a nationwide Danish cohort study. ESC Heart Fail.

[15] Mruma HA, McQuillan R, Norrie J (2020). The association of malaria infection and gestational hypertension in Africa: Systematic review and meta-analysis. J Glob Health.

[16] Barker TH, Stone JC, Hasanoff S, Price C, Kabaghe A, Munn Z (2023). Effectiveness of dual active ingredient insecticide-treated nets in preventing malaria: A systematic review and meta-analysis. PLoS ONE.

[17] Ugwu FSO (2023). Why the World Health Organization should reconsider long lasting insecticide nets (LLIN) and indoor residual spraying (IRS) in primary mosquito/malaria control in favour of house screening. Journal of Biological Research and Biotech.

[18] Amelo W, Makonnen E (2021). Efforts Made to Eliminate Drug-Resistant Malaria and Its Challenges. BioMed Research International.

[19] García GA, Fuseini G, Mba Nlang JA, Nsue Maye VO, Bela NR, Wofford RN (2022). Evaluation of a Multi-Season, Community-Based Larval Source Management Program on Bioko Island, Equatorial Guinea. Frontiers in Tropical Diseases.

[20] Alazwari IAH, Alarsan S, Alkhateeb NA, Salameh EK (2023). Designing Effective Health Education Programs: A Review of Current Research and Best Practices. NAMJ.

[21] Opoku AS, Addison TK, Gebre Y, Mutala AH, Antwi KB, Abbas DA (2023). Accuracy of diagnosis among clinical malaria patients: comparing microscopy, RDT and a highly sensitive quantitative PCR looking at the implications for submicroscopic infections. Malar J.

[22] Schneider ZD, Shah MP, Boily MC, Busbee AL, Hwang J, Lindblade KA, Gutman JR (2024). Mass Drug Administration to Reduce Malaria Transmission: A Systematic Review and Meta-Analysis. The American Journal of Tropical Medicine and Hygiene.

[23] RTS,S Clinical Trials Partnership (2015). Efficacy and safety of RTS,S/AS01 malaria vaccine with or without a booster dose in infants and children in Africa: final results of a phase 3, individually randomised, controlled trial. Lancet.

[24] Seder RA, Chang LJ, Enama ME, Zephir KL, Sarwar UN, Gordon IJ (2013). VRC 312 Study Team: Protection against malaria by intravenous immunization with a nonreplicating sporozoite vaccine. Science.

[25] Tiono AB, Nébié I, Anagnostou N, Coulibaly AS, Bowyer G, Lam E (2018). First field efficacy trial of the ChAd63 MVA ME-TRAP vectored malaria vaccine candidate in 5-17 months old infants and children. PLOS ONE.

[26] Datoo MS, Natama MH, Somé A, Traoré O, Rouamba T, Bellamy D (2021). Efficacy of a low-dose candidate malaria vaccine, R21 in adjuvant Matrix-M, with seasonal administration to children in Burkina Faso: a randomised controlled trial. The Lancet.

[27] Nosten F, Luxemburger C, Kyle DE, Ballou WR, Wittes J, Wah E (1996). Randomised double-blind placebo-controlled trial of SPf66 malaria vaccine in children in northwestern Thailand. The Lancet.

[28] Dejon-Agobe JC, Ateba-Ngoa U, Lalremruata A, Homoet A, Engelhorn J, Nouatin OP (2019). Controlled Human Malaria Infection of Healthy Adults with Lifelong Malaria Exposure to Assess Safety, Immunogenicity, and Efficacy of the Asexual Blood Stage Malaria Vaccine Candidate GMZ2. Clin Infect Dis.

[29] Khan F, Porter M, Schwenk R, DeBot M, Saudan P, Dutta S (2015). Head-to-Head Comparison of Soluble vs. Qβ VLP Circumsporozoite Protein Vaccines Reveals Selective Enhancement of NANP Repeat Responses. PLoS One.

[30] Minassian AM, Silk SE, Barrett JR, Nielsen CM, Miura K, Diouf A (2021). Reduced blood-stage malaria growth and immune correlates in humans following RH5 vaccination. Med (New York).

[31] Dimala CA, Kika BT, Kadia BM, Blencowe H (2018). Current challenges and proposed solutions to the effective implementation of the RTS, S/AS01 Malaria Vaccine Program in sub-Saharan Africa: A systematic review. PLoS ONE.

[32] Chutiyami M, Saravanakumar P, Bello UM, Salihu D, Adeleye K, Kolo MA (2024). Malaria vaccine efficacy, safety, and community perception in Africa: a scoping review of recent empirical studies. Infection.

[33] Oladipo HJ, Tajudeen YA, Oladunjoye IO, Yusuff SI, Yusuf RO, Oluwaseyi EM (2022). Increasing challenges of malaria control in sub-Saharan Africa: Priorities for public health research and policymakers. Annals of Medicine & Surgery.

[34] El-Moamly AA, El-Sweify MA (2023). Malaria vaccines: the 60-year journey of hope and final success—lessons learned and future prospects. Tropical Medicine and Health.

[35] Osoro CB, Ochodo E, Kwambai TK, Otieno JA, Were L, Sagam CK (2024). Policy uptake and implementation of the RTS,S/AS01 malaria vaccine in sub-Saharan African countries: status 2 years following the WHO recommendation. BMJ Glob Health.

[36] Epstein JE, Paolino KM, Richie TL, Sedegah M, Singer A, Ruben AJ (2017). Protection against *Plasmodium falciparum* malaria by PfSPZ Vaccine. JCI Insight.

[37] Roetynck Sophie, ASTMH (2016). “Immunogenicity of ChAd63/MVA ME-TRAP malaria vectored vaccine is not affected by coadministration with routine EPI vaccines in a randomized controlled trial in Gambian infants and neonates”.

[38] Datoo MS, Dicko A, Tinto H, Ouédraogo JB, Hamaluba M, Olotu A (2024). Safety and efficacy of malaria vaccine candidate R21/Matrix-M in African children: a multicentre, double-blind, randomised, phase 3 trial. The Lancet.

[39] White MT, Verity R, Griffin JT, Asante KP, Owusu-Agyei S, Greenwood B (2015). Immunogenicity of the RTS,S/AS01 malaria vaccine and implications for duration of vaccine efficacy: secondary analysis of data from a phase 3 randomised controlled trial. The Lancet Infectious Diseases.

[40] Salamanca DR, Gómez M, Camargo A, Cuy-Chaparro L, Molina-Franky J, Reyes C (2019). Plasmodium falciparum Blood Stage Antimalarial Vaccines: An Analysis of Ongoing Clinical Trials and New Perspectives Related to Synthetic Vaccines. Front Microbiol.

[41] Ayieko C, Ogola BS, Ochola L, Ngwena GAM, Ayodo G, Hodges JS (2017). Interferon-γ responses to Plasmodium falciparum vaccine candidate antigens decrease in the absence of malaria transmission. PeerJ.

[42] Douglas AD, Baldeviano GC, Lucas CM, Lugo-Roman LA, Crosnier C, Bartholdson SJ (2015). A PfRH5-Based Vaccine Is Efficacious against Heterologous Strain Blood-Stage Plasmodium falciparum Infection in Aotus Monkeys. Cell Host Microbe.

[43] Greenwood B, Gaye O, Kamya MR, Kibiki G, Mwapasa V, Phiri KS (2018). Supporting capacity for research on malaria in Africa. BMJ Glob Health.

[44] Head MG, Goss S, Gelister Y, Alegana V, Brown RJ, Clarke SC (2017). Global funding trends for malaria research in sub-Saharan Africa: a systematic analysis. Lancet Glob Health.

[45] Uzochukwu IC, Eleje GU, Nwankwo CH, Chukwuma GO, Uzuke CA, Uzochukwu CE (2021). COVID-19 vaccine hesitancy among staff and students in a Nigerian tertiary educational institution. Ther Adv Infect Dis.

[46] (2021). World malaria report.

[47] World health statistics (2023). Monitoring health for the SDGs, sustainable development goals - World | ReliefWeb.

[48] Willyard C (2022). The slow roll-out of the world's first malaria vaccine. Nature.

[49] Ojakaa DI, Jarvis JD, Matilu MI, Thiam S (2014). Acceptance of a malaria vaccine by caregivers of sick children in Kenya. Malar J.

[50] Asmare G (2022). Willingness to accept malaria vaccine among caregivers of under-5 children in Southwest Ethiopia: a community based cross-sectional study. Malaria Journal.

[51] Rahim S, Ahmad Z, Abdul-Ghafar J (2022). The polio vaccination story of Pakistan. Vaccine.

[52] Mtenga S, Kimweri A, Romore I, Ali A, Exavery A, Sicuri E (2016). Stakeholders' opinions and questions regarding the anticipated malaria vaccine in Tanzania. Malar J.

[53] Mumtaz H, Nadeem A, Bilal W, Ansar F, Saleem S, Khan QA (2023). Acceptance, availability, and feasibility of RTS, S/AS01 malaria vaccine: A review. Immun Inflamm Dis.

[54] McCoy KD, Weldon CT, Ansumana R, Lamin JM, Stenger DA, Ryan SJ (2021). Are malaria transmission-blocking vaccines acceptable to high burden communities? Results from a mixed methods study in Bo, Sierra Leone. Malaria Journal.

[55] Meñaca A, Tagbor H, Adjei R, Bart-Plange C, Collymore Y, Ba-Nguz A (2014). Factors Likely to Affect Community Acceptance of a Malaria Vaccine in Two Districts of Ghana: A Qualitative Study. PLOS ONE.

[56] Alum EU, Ugwu OPC, Obeagu EI, Okon M (2023). Curtailing HIV/AIDS Spread: Impact of Religious Leaders. Newport International Journal of Research in Medical Sciences.

[57] Laurens MB (2019). RTS,S/AS01 vaccine (Mosquirix™): an overview. Hum Vaccin Immunother.

[58] Syed YY (2022). RTS,S/AS01 malaria vaccine (Mosquirix®): a profile of its use. Drugs Ther Perspect.

[59] Maxmen A (2019). First proven malaria vaccine rolled out in Africa — but doubts linger. Nature.

[60] Targett G (2015). Phase 3 trial with the RTS,S/AS01 malaria vaccine shows protection against clinical and severe malaria in infants and children in Africa. Evid Based Med.

[61] Sinnei DK, Karimi PN, Maru SM, Karengera S, Bizimana T (2023). Evaluation of vaccine storage and distribution practices in rural healthcare facilities in Kenya. Journal of Pharmaceutical Policy and Practice.

[62] Pambudi NA, Sarifudin A, Gandidi IM, Romadhon R (2022). Vaccine cold chain management and cold storage technology to address the challenges of vaccination programs. Energy Reports.

[63] Ward K, Stewart S, Wardle M, Sodha SV, Tanifum P, Ayebazibwe N (2019). Building health workforce capacity for planning and monitoring through the Strengthening Technical Assistance for routine immunization training (START) approach in Uganda. Vaccine.

[64] Ansar F, Azzam A, Rauf MS, Ajmal Z, Asad UG, Rauf S (2024). Global Analysis of RTS, S/AS01 Malaria Vaccine Acceptance Rates and Influencing Factors: A Systematic Review. Cureus.

[65] Grant J, Gyan T, Agbokey F, Webster J, Greenwood B, Asante KP (2022). Challenges and lessons learned during the planning and early implementation of the RTS,S/AS01E malaria vaccine in three regions of Ghana: a qualitative study. Malaria Journal.

[66] Guignard A, Praet N, Jusot V, Bakker M, Baril L (2019). Introducing new vaccines in low- and middle-income countries: challenges and approaches. Expert Review of Vaccines.

[67] Olawade DB, Wada OZ, Ezeagu CN, Aderinto N, Balogun MA, Asaolu FT (2024). Malaria vaccination in Africa: A mini-review of challenges and opportunities. Medicine (Baltimore).

[68] Adeyemo AO, Aborode AT, Bello MA, Obianuju AF, Hasan MM, Kehinde DO (2022). Malaria vaccine: The lasting solution to malaria burden in Africa. Ann Med Surg (Lond).

[69] Oduoye MO, Haider MU, Marsool MDM, Kareem MO, Adedayo AE, Abdulkarim AS (2024). Unlocking the potential of novel RTS, S/AS01, and R21/Matrix-M™ malaria vaccines in African nations. Health Science Reports.

